# Recent Uses of Thyroid Preparations

**Published:** 1893-12-02

**Authors:** 


### EDINBURGH ROYAL INFIRMARY.

### Recent Uses of Thyroid Preparations.

Little more than two years have elapsed since Dr. George Murray, of Newcastle, introduced to the profession a method for the treatment of myxœdema by the administration of the thyroid gland of the sheep, and already the literature of the subject is voluminous.

It is to be regretted that the dosage of the remedy has as yet been expressed in fractions of a sheep's thyroid—a somewhat variable quantity for an agent of undoubted activity. It is usually stated at the equivalent of from an eighth to one half a sheep's thyroid twice a week or less frequently.

A number of cases of myxœdema have been thus treated in Edinburgh with success, many of the patients having been exhibited to the Medico-Chirurgical Society at a special meeting to consider the question.* In addition to these, however, the application of thyroid treatment has been extended to cases of a different character with results which seem to promise a success as striking as that which has followed its use in myxœdema. From myxœdema to sporadic cretinism is a natural step, and it might be inferred that a remedy so useful in the former might prove beneficial in the latter. A case reported by Dr. John Thomson† proves this hope to be well founded. A male creten aged 18 years, measuring 33½ inches high, under thyroid treatment for three months, gained over two inches in height, and at the same time his physical and mental condition was remarkably improved. The excellent photographs which accompany the paper bring this out in a striking manner. Since then several other cases have been treated in a similar manner with success. Between myxœdema and psoriasis there seems little connection, but the latter disease has been found in some cases to be susceptible to what we may call the specific treatment of the former. This advance rests on the basis of clinical observation. Dr. Byron Bramwell observed that in cases of myxœdema treated by thyroid gland a profuse desquamation of the skin took place, and happily argued that this action might be utilised in cases of skin disease. In three cases of psoriasis reported by him*, the administration of thyroid in the form of fresh gland or glycerine extract, was followed within a few days by marked improvement, the eruption being detached in large scaly masses, and rapidly disappeared, leaving the skin in a natural condition.

* *Edin. Med. Jour.*, May, 1893.
† *Edinburgh Medical Journal*, May, 1893.

While it is still too early to pronounce a definite verdict on these results, they are sufficient to encourage the further trial of the remedy, and its extension to other diseases of this class, and the effects will be awaited with the greatest interest.

It is not a little remarkable that myxœdema, which was first described as a separate disease by Sir William Gull but twenty years ago, should in so short a time have yielded so many of its secrets as regards its pathology and treatment, and though much has been done by Continental observers, it is creditable to British medicine that the names most closely associated with this condition are those of physicians and physiologists belonging to this country.

It is important also in so far as it has opened up a new field in which, it may be hoped, other remedies may yet be discovered in the form of organic extracts, and forms as it were a type of that medical knowledge, which founded on the evidences of the laboratory, the bed-side, and the post-mortem theatre—not singly, but each corroborating and correcting the other—forms the best basis for treatment.

* *British Medical Journal*, 1893. Vol. ii., p. 933.

